# DoSA: Database of Structural Alignments

**DOI:** 10.1093/database/bat048

**Published:** 2013-07-11

**Authors:** Swapnil Mahajan, Garima Agarwal, Mohammed Iftekhar, Bernard Offmann, Alexandre G. de Brevern, Narayanaswamy Srinivasan

**Affiliations:** ^1^Dynamique des Structures et Interactions des Macromolécules Biologiques (DSIMB), UMR-S INSERM S665, Faculté des Sciences et Technologies, Université de La Réunion, F-97715 Saint Denis Messag Cedex 09, La Réunion, France, ^2^Molecular Biophysics Unit, Indian Institute of Science, Bangalore 560012, India, ^3^Laboratoire d’Excellence, GR-Ex, Paris, F-75739, France, ^4^National Centre for Biological Sciences, Tata Institute of Fundamental Research, UAS-GKVK Campus, Bellary road, Bangalore 560065, India, ^5^Université de Nantes, UFIP CNRS FRE 3478, 2 rue de la Houssinière, 44000 Nantes, France, ^6^INSERM UMR-S 665, DSIMB, Paris, F-75739 France, ^7^Université Paris Diderot, Sorbonne Paris Cité, UMR-S665, Paris, F-75739, France and ^8^Institut National de la Transfusion Sanguine (INTS), Paris, F-75739, France

## Abstract

Protein structure alignment is a crucial step in protein structure–function analysis. Despite the advances in protein structure alignment algorithms, some of the local conformationally similar regions are mislabeled as structurally variable regions (SVRs). These regions are not well superimposed because of differences in their spatial orientations. The Database of Structural Alignments (DoSA) addresses this gap in identification of local structural similarities obscured in global protein structural alignments by realigning SVRs using an algorithm based on protein blocks. A set of protein blocks is a structural alphabet that abstracts protein structures into 16 unique local structural motifs. DoSA provides unique information about 159 780 conformationally similar and 56 140 conformationally dissimilar SVRs in 74 705 pairwise structural alignments of homologous proteins. The information provided on conformationally similar and dissimilar SVRs can be helpful to model loop regions. It is also conceivable that conformationally similar SVRs with conserved residues could potentially contribute toward functional integrity of homologues, and hence identifying such SVRs could be helpful in understanding the structural basis of protein function.

**Database URL:** http://bo-protscience.fr/dosa/

## Introduction

Protein structure comparison is an important step in improving our understanding of the mechanistic basis of function of a protein. Insights on function can be obtained by comparing the structures of proteins of a yet-unknown function with the structures of related proteins of known function (1). Depending on their functional importance and structural roles, different regions of proteins have different levels of evolutionary pressure acting on them. Regions of high evolutionary constraints are usually implicated in maintaining structural or functional integrity of the protein. Such regions are identified in the Conserved Domain Database where protein structures are used to define domain boundaries and provide insights into sequence–structure–function relationships (2). Regions that have low evolutionary constraints or undergo neutral mutations are usually flexible and are manifested as insertions, deletions and substitutions in the alignments (3, 4). Flexibility of these regions can vary from subtle local conformational variation to large changes in orientations of the regions to accommodate insertions (5). These insertions may promote functional diversity by creating a new binding site or by changing a present binding site for ligands or macromolecules (6, 7). Therefore, protein 3D structure comparison becomes an important tool to analyze structural divergence and in turn functional divergence.

Alignment of protein 3D structures is much more complex than protein sequence alignment (8). Many methods have been developed to circumvent the complexities in aligning protein 3D structures, e.g. DALI (9), CE (10), SSAP (11), MAMMOTH (12), COMPARER (13), FATCAT (14), Matt (15) and FlexProt (16). Link outs for these structure alignment methods are provided on the database site under ‘other resources’ tab. From the results of these methods, it is not often clear if a pair of structurally variable regions (SVRs) from the two homologues truly corresponds to different conformations or, although they have similar conformations, they look misaligned because of differences in spatial orientations of these regions. In our previous work (17), we have addressed the above-mentioned problem of identification of conformationally similar local regions that differ in spatial orientations or do not superimpose well. Protein structural alignments were analyzed using a structural alphabet, Protein blocks (PBs) (18–20), which represent local structures that are recurrent in proteins. PBs are the most widely used structural alphabets to date (20, 21). PBs are a set of 16 local protein structures (18). These 16 PBs are the abstraction of local protein backbone structures. Each of the 16 PBs is defined by a vector of eight backbone torsion angles associated with five consecutive residues and represented by the alphabet characters from ‘a’ to ‘p’. Hence, a protein structure can be transformed from a 3D to a 1D sequence of PBs. This ability to represent protein structure in 1D has led to the development of new approaches for protein structure analysis (20).

The Database of Structural Alignments (DoSA) is a result of our previous work on identification of structurally similar SVRs in homologous proteins by using a PB substitution matrix combined with the modified CLUSTALW (22) algorithm [for more details refer to (17)]. In our previous work, we clearly show that optimal residue–residue equivalences could be achieved on the basis of PBs leading to improved local alignments. We also showed that this is particularly useful in comparative modeling of loop regions. Moreover, understanding of sequence–structure relationships can be enhanced through this approach (17).

DoSA provides improved structure-based sequence alignments of homologous proteins especially focusing on the SVRs. This database proposes a refined view of the SVRs, which may contain local similarity concealed in global alignment of homologous protein structures. DoSA provides the unique information about conformationally dissimilar and conformationally similar SVRs in pairwise structural alignments. It gives the refined structural alignment in terms of amino acid sequence, PBs and the 3D superimposition itself; the protein superimposition can be viewed through the Jmol applet (http://www.jmol.org/).

## General features of DoSA:

**Figure 1.**
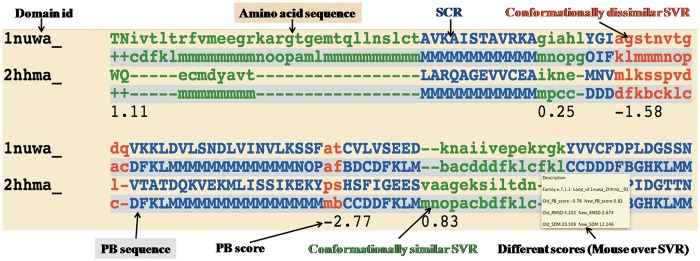
*Example of one representative pairwise structural alignment.* In pairwise structure-based sequence alignments, the two sequences are given as classical sequences alignments. They are identified through their domain ID, the amino acid sequence being the first written, the second line being the PB sequence. SCRs are shown in uppercase and blue. Conformationally similar and dissimilar SVRs are shown in lowercase green and red, respectively. Corresponding PB sequences are shown with a gray background. Under the SVRs are given their personal SVR scores. The different metrics to assess the quality of SVRs are displayed as a mouseover event in a text box.

**Figure 2.**
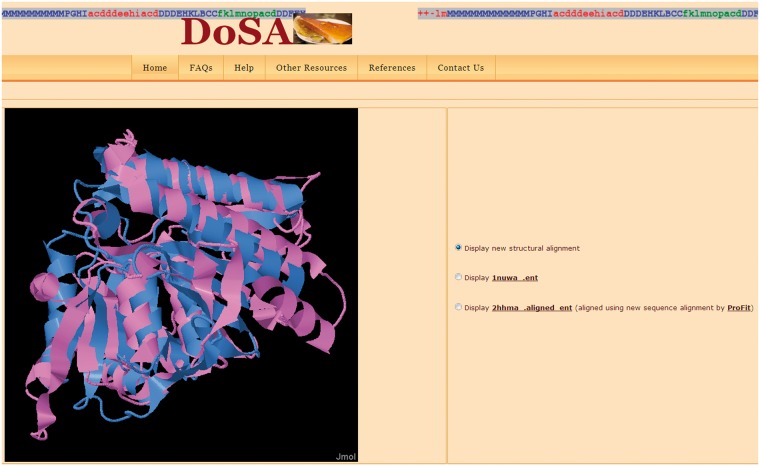
*Visualization of the pairwise structural alignment with the Jmol applet*. The structural alignments are based on improved structure-based sequence alignments, as seen in Figure 1. Users can also view individual protein domain structures using the Jmol applet by clicking on the buttons.

- *Improved structure-based sequence alignments*: Improved pairwise alignments of homologous protein domains from Phylogeny and ALIgnment of homologous protein structures (PALI v2.7) (23) with their corresponding PB sequence alignments are available in DoSA. The improved structure-based sequence alignments and their corresponding PB sequence alignments can also be downloaded as text files. The alignments are categorized according to SCOP (24) families, which are further categorized as *α*, *β*, *α*/*β*, *α* + *β*, small proteins and multi-domain proteins classes.
- *Conformationally similar and dissimilar SVRs*: SVRs in pairwise alignments are highlighted with colors and are shown in lowercase, whereas structurally conserved regions (SCRs) are shown in uppercase. SVRs are color coded green and red representing conformationally similar and dissimilar SVRs, respectively. SCRs are shown in blue (Figure 1). Please note that SVRs at the N and C terminals of alignments were excluded from the analysis because of two unassigned PB positions at the ends of each PB sequence.
- *Different metrics to characterize the quality of superimposition:* A precise definition of the structural similarity is not trivial. So DoSA provides different scores to help the user, as the mouseover event for pairwise alignments, e.g. PB score, root mean square deviation (RMSD) and structural distance metric (SDM) (17, 25, 26) of SVRs before and after realignment are displayed. PB score is based on the use of an exclusive PB substitution matrix (27). This matrix is equivalent to an amino acid substitution matrix for the PBs. PB score is simply the sum of the aligned PBs with this PB substitution matrix. RMSD is the Euclidean distance between the C*α* of the protein fragments. SDM is derived from RMSD, but takes into account the length of the protein fragments compared (25, 26). By using a PB score cutoff of more than or equal to −0.42 [for more details refer to (17)], we can identify regions that are considered as SVR in the PALI database as conformationally similar SVRs, e.g. the SVR was identified as conformationally similar SVR (Figure 1, labeled as conformationally similar SVR), which had an RMSD of 4.2 Å and an SDM of 20.5 before realignment by modified CLUSTALW using the PB substitution matrix but an RMSD of 2.7 Å and an SDM of 12.3 after realignment.
- *Structure visualization*: Improved pairwise structure-based sequence alignments were used to perform a rigid-body superimposition using the McLachlan algorithm (28) as implemented in the program ProFit (Martin, A.C.R., http://www.bioinf.org.uk/software/profit/). These structural alignments can be viewed and analyzed using a Jmol applet (Jmol: an open-source Java viewer for chemical structures in 3D, http://www.jmol.org/) (Figure 2). The aligned coordinate files can also be downloaded as text files.
- *Database searching*: DoSA can be searched by protein domain ID, Protein Data Bank (PDB) ID, protein family name and protein family ID available in PALI v2.7 (23) using the keyword search option.
- *Multiple structure-based sequence alignments*: Even if the focus of our previous study (17) was on pairwise alignments, for each protein family defined by SCOP 1.73, multiple structure-based sequence alignments obtained using MUSTANG (29) and their corresponding multiple PB sequence alignments are also available on the DoSA web site. SCRs and SVRs in multiple structure alignments were identified by MUSTANG using similar C*α*–C*α* distance thresholds of ≤3 Å and >3 Å, respectively. PBs in the SVRs of these alignments provide useful information about local backbone structural similarity or dissimilarity in different domains of the same protein family.

## Database statistics

The protein data set was obtained from the PALI v2.7 database (23), which contains structure-based sequence alignments generated using DALI (9) for protein domain families defined by the SCOP 1.73 database (24). However, DoSA differs from PALI in a number of ways (see below).

DoSA covers 6420 domains divided in 1867 protein domain families in PALI. A total of 62 730 pairwise alignments are featured in DoSA. These pairwise alignments were divided in 542 610 SCRs and 347 062 SVRs (17). SCRs were identified using a C*α*–C*α* distance threshold of ≤3 Å from all pairwise structural alignments in PALI. Similarly, SVRs were identified from all the pairwise structural alignments of homologous proteins from PALI using a C*α*–C*α* distance threshold of >3 Å for stretches of three or more contiguous residues. The 347 062 SVRs correspond to 49% of the alignment positions in the database. These pairwise structural alignments were converted into alignment of PB sequences. PBs have been assigned using in-house software. Regions corresponding to SVRs were identified in PB alignments and were realigned (17) by a modified CLUSTALW (22) algorithm using a recently improved PB substitution matrix (27). Identification of conformationally similar SVRs was based on PB alignment score. A PB alignment score threshold of −0.42 was applied to distinguish between conformationally similar (score more than or equal to −0.42) and conformationally dissimilar SVRs [score less than −0.42, for more details refer to (17)]. In our analysis, a total of 215 920 complete SVRs with more than three aligned PBs were re-aligned by the modified CLUSTALW algorithm optimized for PB sequence alignments. Of these, ∼74% (159 780) were identified as conformationally similar SVRs using the defined PB alignment score cutoff. For 195 730 SVRs with more than three residues, RMSD and SDM (17, 25, 26) were calculated to assess the quality of alignments.

## Access to DoSA

DoSA can be accessed at http://bo-protscience.fr/dosa/. The database site has been optimized for Mozilla Firefox, Google Chrome and Internet Explorer (version 7 or later) web browsers. Improved pairwise and multiple alignments for 6420 domains can be browsed or searched using key words. Key word searching in DoSA can accept protein domain IDs (e.g. 1vpda1), PDB ID (e.g. 1vpd), incomplete or complete protein family IDs (e.g. a.102.1.2 or a.102.) and incomplete or complete protein family names (e.g. hexokinase or kinase) as input. Protein IDs, family IDs and family names should correspond to PALI v2.7. All the improved pairwise structure-based alignments are annotated to describe conformationally similar and dissimilar SVRs (Figure 1). As shown in Figure 1, different scores to characterize the quality of superimposition for the SVRs are available as a mouseover event [see General features of DoSA (iii)]. Pairwise structural alignments can be viewed and analyzed using a Jmol applet (Figure 2). Superimposed coordinate and structure-based sequence alignment with corresponding PB alignment flat files are available to download for all the pairwise structure alignments.

## Discussion

DoSA is complementary to existing structural alignment databases, and it aims at identifying genuine conformationally similar substructures in regions that are otherwise tagged as structurally variable in these databases. This database would hence serve as a valuable resource to study the nature and extent of structural rearrangements in backbone conformations in structural alignments of homologous proteins. DoSA can thus aid in providing clues to model loop regions, for which a homologue of similar length is unavailable (17). The effect of amino acid substitutions on the local structural alterations in the homologous protein structures could also be studied using the structural alignments provided in DoSA. This database can be used to identify equivalent regions in homologous protein structures that do not share structural similarity and in turn to understand the sequence–structure relationships.

It is noteworthy that although DoSA is derived from the PALI database, it is yet different in a number of ways. Most significantly, PALI is broad based with a few general features, whereas DoSA is a structure-based alignment database that specializes on getting clarity on apparent SVRs. DoSA is specialized in identifying SVRs (often loops) with genuine conformational differences and SVRs that are conformationally similar although not superimposable in a global superposition because of rigid-body orientational differences. In the future, DoSA will be updated with the new releases of the PALI database.

## Supplementary Data

Supplementary data are available at *Database* Online.

## Funding

Indo–French collaborative grant (CEFIPRA/IFCPAR 3903-E to N.S., A.G.deB., G.A.); Department of Biotechnology (DBT), Government of India (to N.S.) (in part) and by grants from the Ministry of Research (France); University Paris Diderot (France); National Institute for Blood Transfusion (INTS, France); National Institute for Health and Medical Research (INSERM, France); ‘Investissements d’avenir’, Laboratories of Excellence GR-Ex (to A.G.deB.). A PhD scholarship grant from Fonds Européen de Dévelopment Régional and Conseil Regional de La Réunion (20100079, Tiers: 144645 to S.M.) and the Department of Biotechnology (DBT), Government of India (to G.A.). Université de Nantes, (UFIP CNRS FRE 3478) (to B.O.) (in part). Funding for open access charge: Department of Biotechnology, Government of India.

*Conflict of interest*. None declared.

## Supplementary Material

Supplementary Data
