# Supplementary material for: DoSA: Database of Structural Alignments
Source: Database (Oxford). 2013 Jul 11;2013:bat048. doi: 10.1093/database/bat048 (PMC3708618; doi:10.1093/database/bat048)
Supplement: Supplementary Data [file supp_2013_bat048_index.html]

DoSA: Database of Structural Alignments — Supplementary Data 

# DoSA: Database of Structural Alignments

## 

files

**Files in this Data Supplement:**

- Supplementary Data - doc file
