# Supplementary material for: DoSA: Database of Structural Alignments
Source: Database (Oxford). 2013 Jul 11;2013:bat048. doi: 10.1093/database/bat048 (PMC3708618; doi:10.1093/database/bat048)
Supplement: Supplementary Data [file supp_bat048_DoSA_Supplementary_data_1.doc]

**Supplementary data 1**

**DoSA: Database of Structural Alignments**

Swapnil Mahajan, Garima Agarwal, Mohammed Iftekhar, Bernard Offmann, Alexandre G. de Brevern and Narayanaswamy Srinivasan.

## Comparison of alignment quality

We compared the PB scores of realigned-SVRs from DoSA database with scores obtained by re-aligning same SVRs using two other structural alignment methods namely, MUSTANG (1) and FATCAT (2). We randomly selected 100 SVR pairs for this comparison. Out of these 100 SVR pairs MUSTANG and FATCAT algorithms were able to align 81 and 71 SVR pairs respectively. Alignments provided by these algorithms were converted into PB sequence alignments and PB scores were calculated for these SVR pairs. The scatter plots of distribution of PB scores from DoSA alignments against MUSTANG PB scores and FATCAT PB scores are shown in supplementary figures S1 and S2 respectively.

Total of 55 SVR pairs, out of 81, have better PB scores in DoSA alignments compared to the scores corresponding to the alignments generated by MUSTANG. Similarly, 41 SVR pairs, out of 71, have better PB scores in DoSA alignments compared to the scores corresponding to the alignments generated by FATCAT.


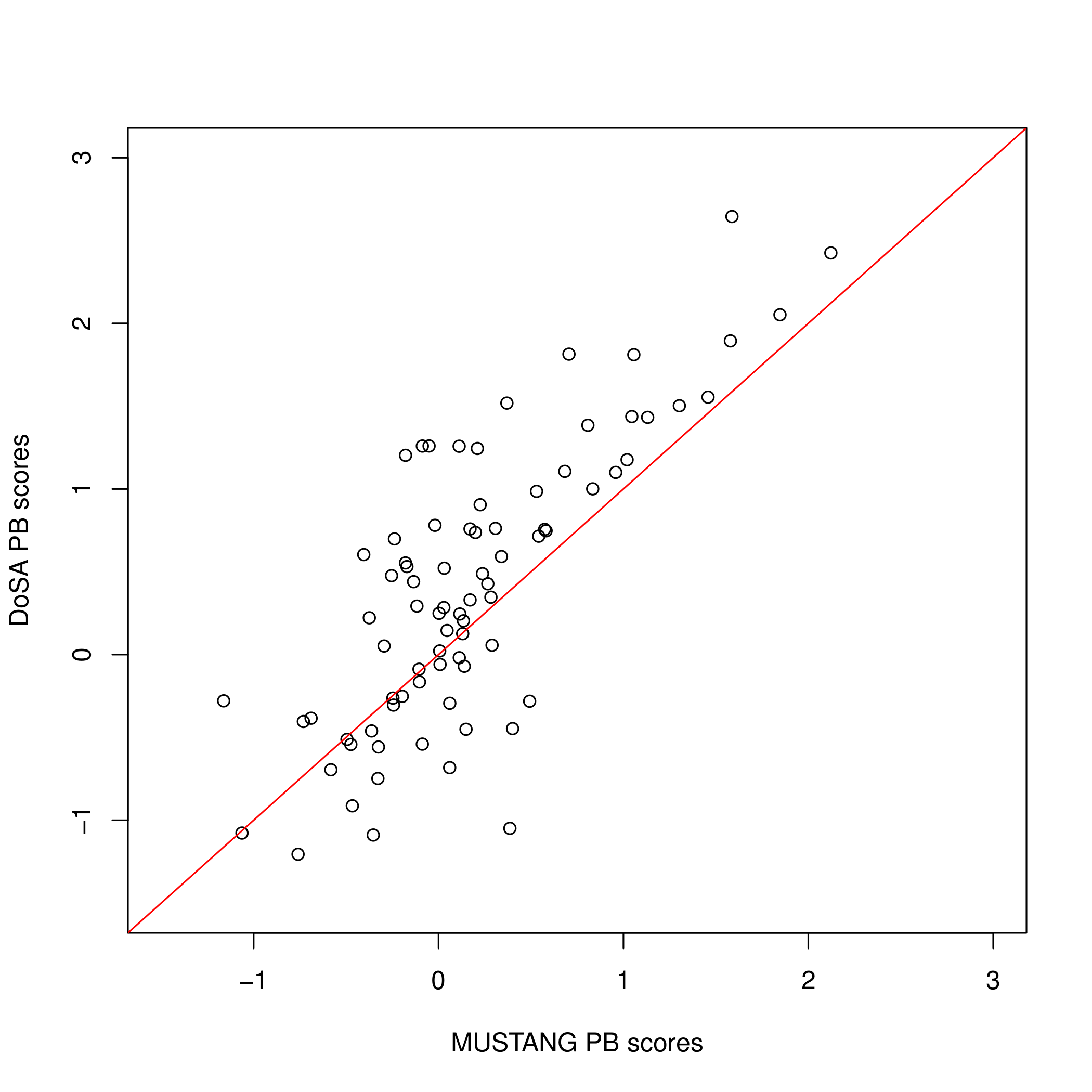


Figure S1: *Comparison of PB scores of alignments of 81 SVR pairs using DoSA and MUSTANG algorithms. The red line corresponds to 45 degrees line with X and Y-axis. For a majority of the cases PB scores as provided by DoSA is better than that from MUSTANG.*


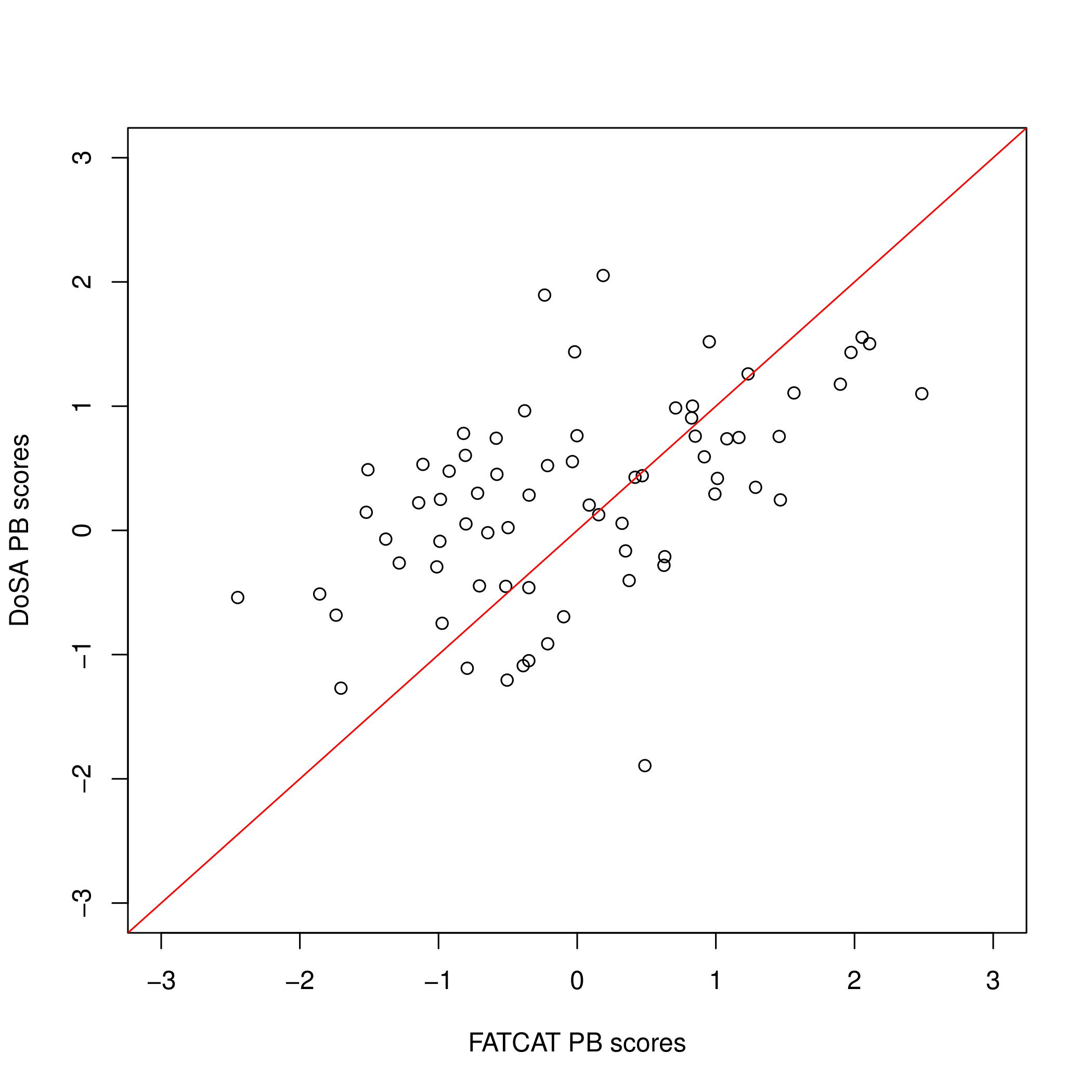


Figure S2: *Comparison of PB scores of alignments of 71 SVR pairs using DoSA and FATCAT algorithms. The red line corresponds to 45 degrees line with X and Y-axis. For a majority of the cases PB scores as provided by DoSA is better than that from FATCAT.*

## References

1. Konagurthu,A.S., Whisstock,J.C., Stuckey,P.J. and Lesk,A.M. (2006) MUSTANG: a multiple structural alignment algorithm. *Proteins*, **64**, 559–574, 10.1002/prot.20921.
2. Ye,Y. and Godzik,A. (2003) Flexible structure alignment by chaining aligned fragment pairs allowing twists. *Bioinformatics*, **19 Suppl 2**, ii246-255.
